# BioLINK Special Interest Group Session on the Future of Scientific Publishing

**DOI:** 10.1371/journal.pcbi.1000398

**Published:** 2009-05-29

**Authors:** Scott Markel

**Affiliations:** Accelrys (SciTegic R&D), San Diego, California, United States of America; University of California San Diego, United States of America

Scott Markel is Vice President, International Society for Computational Biology (ISCB); Co-Chair, ISCB Publications Committee; and Associate Editor, *PLoS Computational Biology*.

The BioLINK SIG meeting has been regularly held in association with the ISMB conference (Intelligent Systems for Molecular Biology—the annual conference of the International Society for Computational Biology) since 2001, focusing on the development and application of resources and tools for biomedical text mining. The SIG (Special Interest Group) is interdisciplinary in nature, and brings together researchers applying natural language processing, text mining, and information extraction and retrieval in the biomedical domain with scientists from bioinformatics and biology. This year's meeting at the combined ISMB/ECCB (European Conference on Computational Biology) conference in Stockholm includes two new sessions, one dedicated to extraction of information from images, and one devoted to the future of scientific publishing. The publishing session, co-organized by BioLINK with the collaboration of the ISCB Publications Committee (http://www.iscb.org/iscb-leadership-a-staff-/117) and *PLoS Computational Biology* (http://www.ploscompbiol.org), has been added in response to the very favorable reviews of last year's Special Session on the same topic. The session format has been expanded to two two-hour segments, both of which will be open to ISMB conference registrants. The first segment will feature scientific presentations from David Shotton, Anita de Waard, Dietrich Rebholz-Schuhmann, and Philip E. Bourne. The second segment will include presentations from journal publishers and will finish with an open discussion.

## “Adventures in Semantic Publishing: Exemplar Semantic Enhancements of a Research Article”

### David Shotton (University of Oxford)

Last summer, we undertook manual semantic enhancements to a biomedical research article, providing enrichment to its content and increased access to datasets within it, to provide a compelling existence proof of the possibilities of semantic publication (http://dx.doi.org/10.1371/journal.pntd.0000228.x001). These semantic enhancements include provision of live DOIs and hyperlinks; semantic markup of textual terms with links to relevant third-party information resources; interactive figures; a reorderable reference list; a document summary containing a study summary, a tag cloud, and a citation analysis; and two novel types of semantic enrichment: the first a Supporting Claims Tooltip to permit “Citations in Context”, and the second Tag Trees that bring together semantically related terms. In addition, we published downloadable spreadsheets containing data from within tables and figures, enriched these with provenance information, and demonstrated various types of data fusion (mashups) with results from other research articles and with Google Maps. We also published machine-readable RDF metadata both about the article and about the references it cites, for which we developed a Citation Typing Ontology, CiTO (http://purl.org/net/cito/).

In my presentation, I will explain what we achieved by means of a live link to the online enhanced paper, discuss the significance of this work in terms of recent developments in automated text mining, and consider the future of semantic publishing as part of mainstream research journal production workflows. My aim is to excite the imaginations of researchers and publishers, stimulating them to explore the possibilities of semantic publishing for their own research articles, and thereby break down present barriers to the discovery and reuse of information within traditional modes of scholarly communication.

## “From Proteins to Hypotheses—Some Experiments in Semantic Enrichment”

### Anita de Waard (Elsevier Labs, Amsterdam, and Utrecht Institute of Linguistics, Utrecht University)

I will discuss a number of initiatives in which I am involved to improve and enhance access to scientific knowledge from collections of research articles. First, at Elsevier Labs, we added manually annotated Structured Digital Abstracts in *FEBS Letters* articles (http://www.febsletters.org/content/sda_summary) containing curated data on protein–protein interactions. To help authors identify these, within the OKKAM EU Project we are creating a Word plug-in using text mining technologies connected by a Web Service to the authoring environment. Second, I will discuss work at Utrecht University regarding scientific discourse analysis, focusing on the identification of different cognitive realms (experiments and conceptual models) in a full-text research publication, and the linguistic methods by which authors identify the epistemic (“truth value”) status of statements. I will then discuss some collaborative efforts for the creation of a common framework to bootstrap efforts in this area. Last, I will describe efforts at Elsevier Labs and the University of Utrecht to stimulate and contribute to the discussion on changing models of publishing. We organized the Elsevier Grand Challenge (http://www.elseviergrandchallenge.com/) to help stimulate collaboration with researchers interested in addressing the redefinition of scientific communication, and I will discuss some future plans.

## “ELIXIR Scientific Literature Interdisciplinary Interactions”

### Dietrich Rebholz-Schuhmann (European Bioinformatics Institute)

Scientific literature is nowadays distributed in electronic form through online Web portals. ELIXIR Work Package 8 (WP8; http://www.elixir-europe.org/page.php?page=wp8) analyzes the academic and commercial stakeholders' needs for automatic exploitation of the resources.

Scientific literature is kept in national and international repositories that currently still lack connectivity. The biomedical community is driven by the idea of the integration of all data resources (including literature) from the level of molecular biology to medicine, leading to multidisciplinary research. The appropriate infrastructure and tools need to be in place to facilitate full exploitation of the literature across scientific domains and at various levels of end user expertise. Scientific literature is unstructured in contrast to the scientific databases. This has led to (1) the development of text mining and knowledge discovery solutions that recover facts from the scientific literature, (2) curation efforts to include scientific facts into the main databases, and (3) efforts around various wiki-like projects to produce annotations. The exploitation of the scientific literature has to (1) fulfill multidisciplinary needs, (2) exploit ontological resources (Semantic Web approaches), (3) deliver enhanced digital content, and (4) follow standards for efficient integration.

## “OpenID vs. ResearcherID”

### Philip E. Bourne (University of California San Diego)

Scientists (at least their profiles) and their scholarly output exist in cyberspace, but the relationship between the two is far from established. Scientists may not be identified uniquely, and much of their output is not easily referenced. The Digital Object Identifier (DOI) was a big step in uniquely identifying a scientific journal publication, and has been embraced by the majority of publishers. I would argue that the time is here for extending this scheme to uniquely identify scientists (authors) with all their respective scholarly output. This is much more than traditional journal publications, and includes database depositions, reviews for grants and journals, blog postings: in fact, anything they wish to have uniquely associated with their name. I will discuss efforts in this direction and what I think it will take to really make such a scheme work—a scheme that starts with the publishers.

The publishers' panel will follow the scientific presentations. The publishers will be free to comment on the presentations or to address other topics, such as validation processes and quality measures (e.g., the future of the peer review model, alternatives to impact factors), dissemination (e.g., open-access models), and discoverability (e.g., linking, applying new technologies). Confirmed participants include Claire Bird (Oxford University Press), Mark Patterson (Public Library of Science), Matt Day (*Nature*), Robert Campbell (Wiley-Blackwell), Matt Cockerill (BioMed Central), and David Tranah (Cambridge University Press).

The BioLINK SIG meeting will be held at the Stockholm ISMB/ECCB 2009 meeting on Sunday and Monday, the 28^th^ and 29^th^ of June. The Future of Scientific Publishing session will take place in the afternoon of Monday, the 29^th^ of June. See http://www.cs.queensu.ca/biolink09 for additional details.

